# The Experimental Design Assistant

**DOI:** 10.1371/journal.pbio.2003779

**Published:** 2017-09-28

**Authors:** Nathalie Percie du Sert, Ian Bamsey, Simon T. Bate, Manuel Berdoy, Robin A. Clark, Innes Cuthill, Derek Fry, Natasha A. Karp, Malcolm Macleod, Lawrence Moon, S. Clare Stanford, Brian Lings

**Affiliations:** 1 National Centre for the Replacement, Refinement and Reduction of Animals in Research (NC3Rs), London, United Kingdom; 2 Certus Technology Associates Ltd, Exeter, United Kingdom; 3 GlaxoSmithKline, Stevenage, United Kingdom; 4 University of Oxford, Oxford, United Kingdom; 5 Envigo, Alconbury, United Kingdom; 6 University of Bristol, Bristol, United Kingdom; 7 University of Manchester, Manchester, United Kingdom; 8 Wellcome Trust Sanger Institute, Hinxton, United Kingdom; 9 Quantitative Biology IMED, AstraZeneca R&D, Cambridge, United Kingdom; 10 Centre for Clinical Brain Sciences, University of Edinburgh, Edinburgh, United Kingdom; 11 King’s College London, London, United Kingdom; 12 University College London, London, United Kingdom

## Abstract

Addressing the common problems that researchers encounter when designing and analysing animal experiments will improve the reliability of in vivo research. In this article, the Experimental Design Assistant (EDA) is introduced. The EDA is a web-based tool that guides the in vivo researcher through the experimental design and analysis process, providing automated feedback on the proposed design and generating a graphical summary that aids communication with colleagues, funders, regulatory authorities, and the wider scientific community. It will have an important role in addressing causes of irreproducibility.

## Introduction

The poor reproducibility of findings from animal research has received much attention over the last few years [1], not least because of the impact it has on translation, scientific progress, and the use of resources. It has been estimated that over half of preclinical research is irreproducible (see [2]). There are many reasons for this, aside from the complication that reproducibility can be defined in different ways [3], but flawed experimental design, inappropriate statistical analysis, and inadequate reporting have been flagged as major concerns [4–6]. There is considerable scope for improving the way animal research is designed, conducted, analysed, and reported.

As a starting point, the UK National Centre for the Replacement, Refinement and Reduction of Animals in Research (NC3Rs) developed the Animal research: Reporting of in vivo experiments (ARRIVE) guidelines to improve the reporting of animal experiments [7,8]. As of 2016, compliance with these guidelines was recommended by over 600 leading journals in the biomedical sciences [9]; with more advocating their use each year, this number has now increased to over 1,000 [7]. Compliance should ensure that published articles contain sufficient information to assess the reliability of the findings and enable the experiments to be adequately replicated [8]. Improved reporting should also increase the quality of retrospective studies, such as systematic reviews. However, in order to increase the reliability of findings, the design, conduct, and analysis of individual experiments needs to be improved. Here, we present the Experimental Design Assistant (EDA), which was launched to support the scientific community with this process [10,11].

The EDA (https://eda.nc3rs.org.uk) is a web-based application with an integrated website, which guides researchers through the process of designing animal experiments; the output includes a diagram that improves the transparency of the experimental plan. The resource is freely available and was developed by the authors as an NC3Rs-led collaboration between in vivo researchers and statisticians from academia and industry and a team of software designers who specialise in innovative solutions for the life sciences (http://www.certus-tech.com/). The EDA enables researchers to build a stepwise, schematic representation of an experiment—the EDA diagram—and uses computer-based logical reasoning to provide feedback and advice on the experimental plan. The system’s main features are presented in Table 1.

**Table 1:** Features of the Experimental Design Assistant (EDA).

Features of the EDA include the following:
• A computer-aided design tool to develop a diagram representing the experimental plan,
• feedback from an expert system on the experimental plan (the Critique),
• Analysis Suggestion,
• sample size calculation,
• randomisation sequence generation,
• support for allocation concealment and blinding,
• web-based resources to improve knowledge of experimental design and analysis.

### The EDA improves the reliability of experimental results and analysis

The majority of published in vivo research provides no indication that basic precautions have been taken to obtain reliable findings [4,12]. High internal validity can only be achieved by minimising systematic bias so that observed differences can be confidently associated with the treatment of interest. For example, many publications include no information on randomisation and blinding [4,12]. This is not considered to be just a reporting issue; studies have shown that publications of animal experiments that report the use of such measures also tend to report lower effect sizes compared to those that do not [13]. This implies that experiments are generally not designed and conducted to the highest standards, and the results may not be reliable. Random allocation and blinding have 2 benefits: first, they help meet a key assumption of the statistical analysis, namely, that different groups are drawn from the same background population using random sampling. Second, applying these techniques minimises systematic differences between the treatment groups during the conduct of the experiment, assessment of the results, and data analysis. Such differences can be caused by researchers subconsciously influencing the animals’ allocation to treatment groups, the animals’ behaviour [14], or the handling of the data (e.g., removal of outliers).

Another common concern regarding the reliability of animal experiments is that they are underpowered, using too few animals to yield dependable results. Button, Ioannidis and colleagues [15] estimated the average power in neuroscience animal studies to be around 20%. This constitutes a high risk of missing a genuine effect (a false negative), because only 1 in 5 experiments would have a chance of detecting an effect of the magnitude reported. Conversely, the use of sample sizes that are too small also reduces the reliability of the conclusions from an individual experiment and of the published literature as a whole. When a statistically significant effect is detected, it is less likely to be genuine and its magnitude more likely to be overestimated [15,16]. Indeed, justification for the choice of sample sizes is rarely included in published papers [4,12].

**Fig 1:**
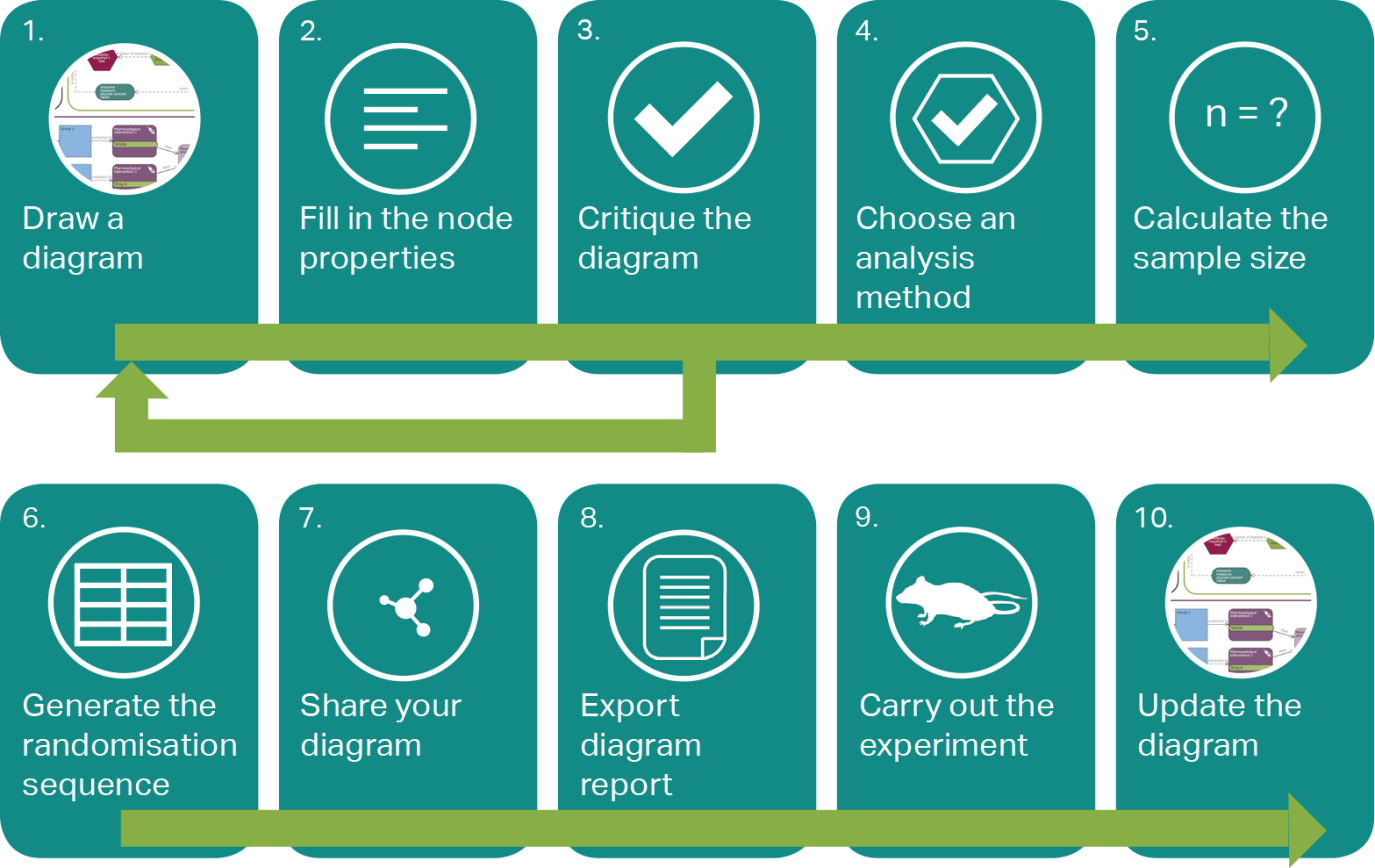
The Experimental Design Assistant (EDA) workflow. The workflow is not fixed and different users might prefer to do some steps in a different order. A potential workflow using the different functionalities of the EDA is described as follows: (1) The user starts by drawing a diagram (with nodes and links) representing the experiment they are planning. Assistance is provided in the form of examples, templates, and video tutorials. (2) Information is added into the node properties, providing more details about the specific step of the process represented by each node. (3) The “Critique” functionality (see Table 2) enables the researcher to obtain feedback on the diagram and the design it represents. The feedback might prompt a change in their plans or the addition of missing information. This is an iterative process and the user might go through the first 3 steps a number of times. (4) Once feedback from the critique has been addressed and the user is satisfied with the final design, the analysis method suggested by the system can be reviewed (see Table 2). (5) Depending on how the data will be analysed, a suitable sample size can be calculated using one of the calculators provided within the system. (6) Once the number of animals needed per group is known, the EDA can generate the randomisation sequence. The spreadsheet detailing the group allocation for each animal can be sent directly to a third party nominated by the user, thus blinding the allocation. This enables the researcher to remain unaware of the group allocation until the data have been collected and analysed. (7) Diagrams can be safely shared with colleagues and collaborators at any stage of the process. (8) The user can export a PDF report, which contains key information about the internal validity of the experiment, a summary of the feedback from the system, and the EDA diagram itself. This report can be submitted as part of a grant application, as part of the ethical review process, or, later on, with a journal manuscript. Alternatively, the diagram data can be exported (as an.eda file) and saved locally or used to register the protocol before the experiment is conducted. (9) Once the planning is complete, the experiment is carried out. (10) The diagram can be updated after data collection to enable the user to keep an accurate record (e.g., to record the number of animals analysed if some failed to complete the experiment or if data are missing for other reasons).

The EDA helps avoid such pitfalls when designing in vivo experiments and improves the reliability of the results—and ultimately, their reproducibility. The system generates a randomisation sequence for the experiment, which takes into account any blocking factors included in the design and provides dedicated functionalities, such as support for blinding and sample size calculation, to assist researchers in following best practice (see Fig 1). A tailored critique provides suggestions on optimising the experimental plan. For example, it helps researchers to identify variables that could confound the outcome and provides advice on how to include them in the randomisation and the statistical analysis. Finally, once the researcher has addressed the feedback and is satisfied with the design, the system advises on which methods of statistical analysis are most appropriate. Designing experiments with the EDA encourages researchers to consider the sources of bias at the design stages of the experiment before the data are collected, ensuring a rigorous design that is more likely to yield robust findings that can be reproduced. The EDA can also be used as a teaching resource, thereby promoting a better understanding of the principles of experimental design at an early stage of the research training process. The process of building an EDA diagram familiarises students with the different components of the design and how they are connected. The visual representation of abstract concepts, such as the experimental unit, the independent variables, or data transformations, brings clarity that enables a detailed discussion of the experimental plan.

### The EDA offers a new standard notation for describing experiments

**Fig 2:**
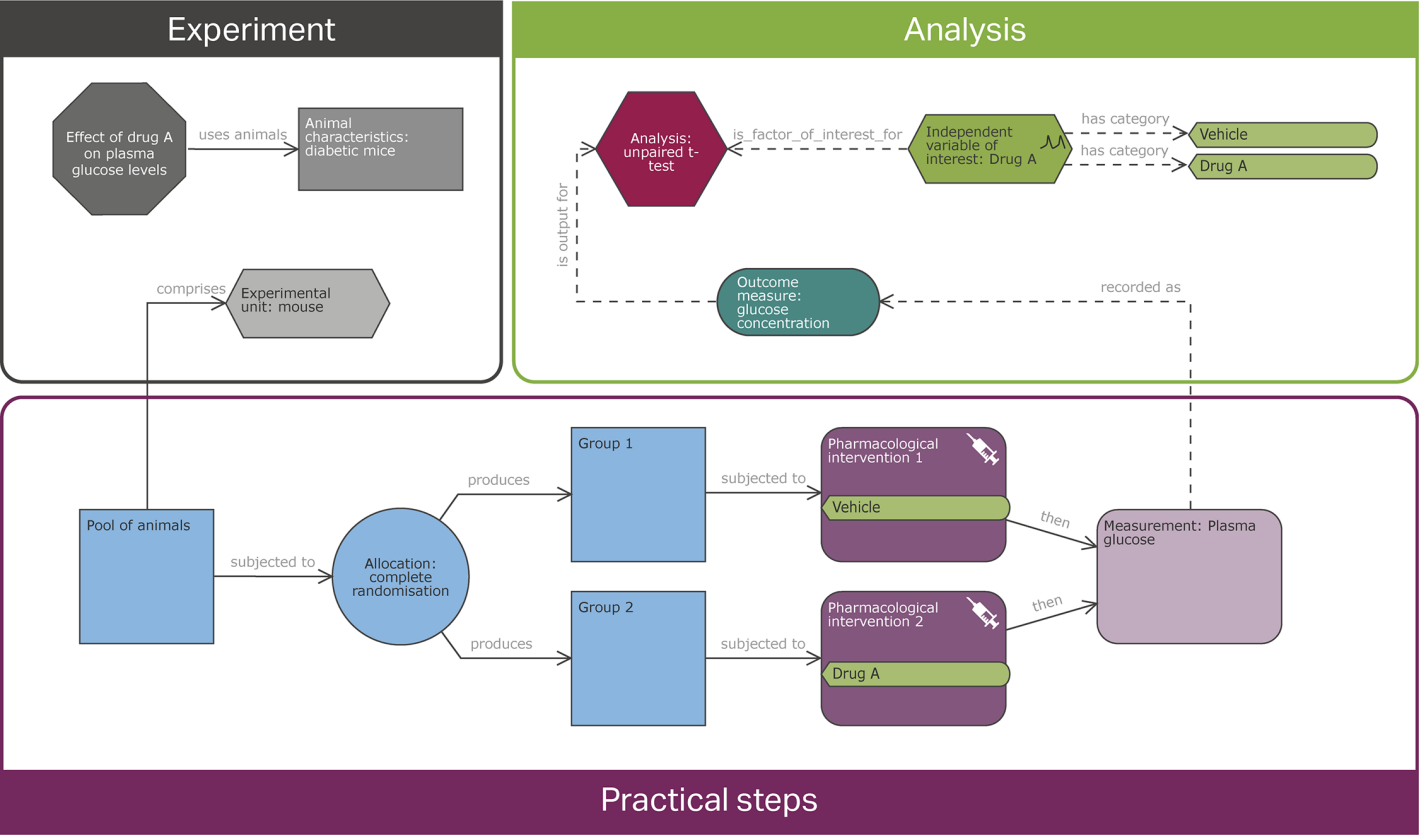
Example of an Experimental Design Assistant (EDA) diagram. EDA diagrams are composed of nodes and links to represent an entire experimental plan. Each node contains properties where specific details are captured (properties are not shown in this picture, but in the EDA they are accessible by clicking on the specific node). This particular example is a simple 2-group comparison. The grey nodes contain high-level information about the experiment, such as the null and alternative hypotheses, the effect of interest (via the experiment node), the experimental unit, or the animal characteristics. The blue and purple nodes represent the practical steps carried out in the laboratory, such as the allocation to groups (allocation node) and the group sizes and role in the experiment (group nodes), the treatments (via the intervention nodes), and the measurements taken (measurement nodes). The green and red nodes represent the analysis, the outcome measures, and the independent variables.

It is difficult to find a technical discipline that has not adopted a schematic, diagrammatic, or symbolic notation to improve communications and the recording of methodological detail. However, there are no universally accepted standards to describe the different components of an experimental design. Different terms can be used to describe the same things; for example, the outcome measure is also known as the dependent variable, the response variable, the outcome variable, or the variable of interest, which can easily be confused with the independent variable of interest (also known as the factor of interest or the predictor of interest). By contrast, the same terms can be used to describe different settings; for example, a ‘repeated measure design’ can imply a situation in which animals receive multiple treatments in a different order (sometimes described as a crossover design). However, it could also refer to a situation in which the response to a given treatment in each animal is measured over time or to a situation in which multiple responses are measured for each animal [17]. The EDA resolves this problem by helping the user generate unambiguous representations of these different designs using EDA diagrams (see Fig 2 and S1 Fig) and hence does not require knowledge or understanding of labels such as ‘repeated measure design’.

A central feature of the EDA is the development of standards for communicating experimental design. This has required the careful definition of the concepts used in experimental design together with an associated vocabulary of preferred terms. This was developed using an iterative approach and tested using a wide range of experimental plans from the published literature. The result is an ontology that supports the capture of every element of an experimental plan, from high-level information such as the hypotheses, effect of interest, and animal characteristics, to the practical steps carried out in the laboratory, as well as details on the variables included in the design and statistical analysis. This ontology has underpinned the development of a computer-aided design tool to support the experimental design process. The tool helps users develop EDA diagrams consistent with the ontology. The diagrams are unambiguous and more explicit than the text descriptions normally included in grant applications or journal publications. Novel ideas and intellectual property contained within the diagrams are protected; a summary of the security measures in place is included on the website: https://eda.nc3rs.org.uk/security. EDA diagrams are also computer interpretable, allowing automated critiquing of designs against recognised best practice without constraining the experimental design process or the plans themselves.

### The EDA enables an effective assessment of the experimental plans

For researchers who have limited access to statistical support, the critical feedback provided by the EDA will be particularly pertinent, as it provides users with information that is specific to the experiment they are planning. The system is not designed to replace specialist statistical advice but can facilitate it. The process of building a design using the EDA emulates the initial fact-finding discussion a researcher might have with a statistician. It helps the researcher to identify much of the information that a statistician would need in order to provide expert advice and presents it in an explicit and standardised format, which can be made available to funding bodies, ethical review committees, journal editors, and peer reviewers.

Users also have the option to share their designs with team members and collaborators, and anecdotal evidence shows that diagrams are extremely useful when discussing the experimental plans within a research team. This is partly because the visual representation enables an efficient critical appraisal of the plans, such as questioning the role of and need for each experimental group, defining variables, identifying potential sources of bias, or debating the type of outcome measures. This detailed scrutiny before the experiment is carried out, or even before the plans are reviewed beyond the laboratory, enables researchers to identify potential pitfalls based on what they know about the science and experimental environment and perhaps follow up with more advanced questions to a statistician.

In addition to problems with the design, analysis, and reporting of scientific research, there are 2 practices that are widely encountered and further compromise the reliability of published results: ‘p-hacking’ (running multiple statistical tests on the same data and choosing the one with the lowest *p* value) and selective outcome reporting (measuring different outcomes, or the same outcome in different ways, and only reporting the ones that reach statistical significance) [18]. These issues effectively represent a post hoc choice of outcome and analysis plan and would be prevented by formalising a clear protocol and plan for the statistical analysis before collecting the data. EDA diagrams are ideal for this purpose. EDA diagrams and the nonvisual information they contain (e.g., prespecified primary outcome measure, chosen method of randomisation) can be registered before the experiment is carried out, on specific platforms such as the Open Science Framework (https://osf.io/) or more generic platforms such as Figshare (https://figshare.com). This provides evidence that a study was planned as it has been reported and confirms that the primary outcome measure has not been changed during the course of the experiment and that any additional results reported should be treated with caution.

### The EDA promotes better understanding of experimental design and analysis

The EDA is not a ‘black box’ that instructs researchers on what design they should use. Instead, it promotes better understanding of experimental design and raises awareness about problems caused by a lack of randomisation and blinding, underpowered experiments, or inappropriate statistical analysis. The feedback provided by the system (see Table 2) enables users to learn about the implications of different design choices and helps them make informed decisions about the most appropriate one to adopt.

**Table 2:** Feedback provided by the Experimental Design Assistant (EDA).

Aspect of experimental design covered by the feedback rules	High-level description of the type of feedback that the EDA can provide
Objective	Provides guidance to identify the null and alternative hypothesis, the effect of interest, and the effect size that is biologically relevant.
Randomisation	Detects when the allocation method is not specified. Highlights the importance of adequate randomisation, advises on randomisation procedures, and prompts users to consider different types of randomisation.
Blinding	Detects when there is no provision for blinding, explains why allocation concealment and blinding are important and the different stages of the experiment that can be blinded, and provides ways blinding can be achieved.
Groups and sample size	Provides guidance to identify the experimental unit(s) and determine suitable sample sizes.
Outcome measures	Highlights the implications of working with continuous and categorical data. Prompts user to identify the primary outcome measure.
Independent variables of interest	Detects when independent variables have not been identified or when they should be treated as continuous or categorical or as repeated factors. Detects when independent variables may be confounded.
Nuisance variables	Advises about nuisance variables commonly seen in animal experiments and how to account for them in the randomisation and analysis.
Statistical analysis	Suggests statistical analysis methods compatible with the design, along with software that can be used to perform these tests. Advises about parametric assumptions and data transformation. Suggests when the advice of a statistician should be sought.

The expert system provides helpful critical feedback to the user based on a set of rules. At the time of writing this article, approximately 140 feedback rules have been implemented; this number will increase over time, improving the precision of the feedback and the ability of the EDA to detect more subtle issues that could be addressed to optimise the design. The EDA feedback is split into 2 distinct functionalities: the Critique and the Analysis Suggestion.

The Critique helps the user build their diagram and identifies missing information and problems with internal consistency. It also suggests improvements to the design and assists the user in identifying and characterising the independent variables in the analysis. Feedback rules are devised to highlight the implications of different design choices, thus enabling researchers to make informed decisions. When an issue is detected (e.g., because additional information is requested or the information provided is not consistent with good practice), an error, warning, or advice flag is placed on a specific node in the diagram. Clicking on the flag opens a pop-up window explaining what the issue is and providing advice on how to address it. Not all EDA users will trigger all feedback rules; for instance, experienced researchers who initially input an adequate level of information within the diagram will see few, if any, prompts.

The Analysis Suggestion can be used after the Critique feedback has been addressed. The information provided is based on the number and type of independent variables of interest, nuisance variables, and outcome measures included in the analysis.

The development of the rule set was informed by workshops in which EDA diagrams representing examples of flawed experimental designs were analysed. These sessions identified, in particular, what information a statistician would require in order to offer the best advice on the design and what advice the statistician would feed back to the researcher to help them identify the information requested by the EDA or to help them improve the design. More information about the development of the feedback feature can be found here: https://eda.nc3rs.org.uk/feedback

Animal studies often use suboptimal statistical analysis, such as failing to use factorial or block designs when appropriate, treating repeated measures as being independent, or failing to account for multiple testing [4,19–22]. The EDA encourages scientists to spend time planning their experiments and optimising the design interactively. It prompts researchers to evaluate carefully their experimental plan and to consider the data to be collected. It also helps to identify sources of variability by providing examples that are commonly encountered in animal research, such as the day of the experiment (if animals are used over several days); the time of day when the experiment is performed; the piece of equipment used to record measurements; the litter or cage mates; the location of cages in the room; or baseline variables, such as the animals’ weight or locomotor activity. Such sources of variability, termed ‘nuisance variables’, can then be accounted for in the design and analysis of the experiment (e.g., as covariates or blocking factors), or they can be standardised. The choice depends on the characteristics of the specific variable, for example, whether it can be treated as a continuous or categorical variable, and the extent of its likely impact on the variability of the response and on how far the conclusions of the experiment can be generalised. It also helps the user to identify independent variables that are repeated factors and warrant a repeated measure analysis, thereby ensuring that users are provided with enough information to avoid common pitfalls.

## Conclusion

The EDA is a novel tool bringing together machine-readable flow diagrams and computer-based logical reasoning to assist the robust and reproducible design of animal experiments. It ensures that the experimental plans are explicit and transparent, thus allowing greater scrutiny before and after data are collected and a meaningful dialogue between researchers and statisticians. It encourages improvements on the design by providing researchers with critical feedback and targeted information. Future development of the system will continue to incorporate user feedback to ensure that the EDA continues to support the needs of the research community. Together with comprehensive reporting and a better understanding of the factors that impact on the reliability and integrity of research findings, the EDA forms part of the solution identified by NC3Rs and others to improve the quality of animal research.

## Supporting information

S1 Fig Here, we present 4 Experimental Design Assistant (EDA) diagrams depicting ‘repeated measure designs’. Diagram 1 shows an experiment in which animals receive multiple treatments in a different order for each animal, and each animal is used as its own control. Diagram 2 shows an experiment in which different groups of animals receive different treatments, 1 treatment per group, and the response to these treatments is measured over time, with each time point included in the analysis. Diagram 3 shows an experiment in which different groups of animals receive different treatments, 1 treatment per group, and the response to these treatments is measured over time, but a summary measure is taken for each animal. Diagram 4 shows an experiment in which multiple responses are measured for each animal.
(PDF)

## Abbreviations

EDA: Experimental Design Assistant
NC3Rs: UK National Centre for the Replacement, Refinement and Reduction of Animals in Research
ARRIVE: Animal research: Reporting of in vivo experiments
