# Supplementary figures and images for: The Experimental Design Assistant
Source: PLoS Biol. 2017 Sep 28;15(9):e2003779. doi: 10.1371/journal.pbio.2003779 (PMC5634641; doi:10.1371/journal.pbio.2003779)

Diagram 1

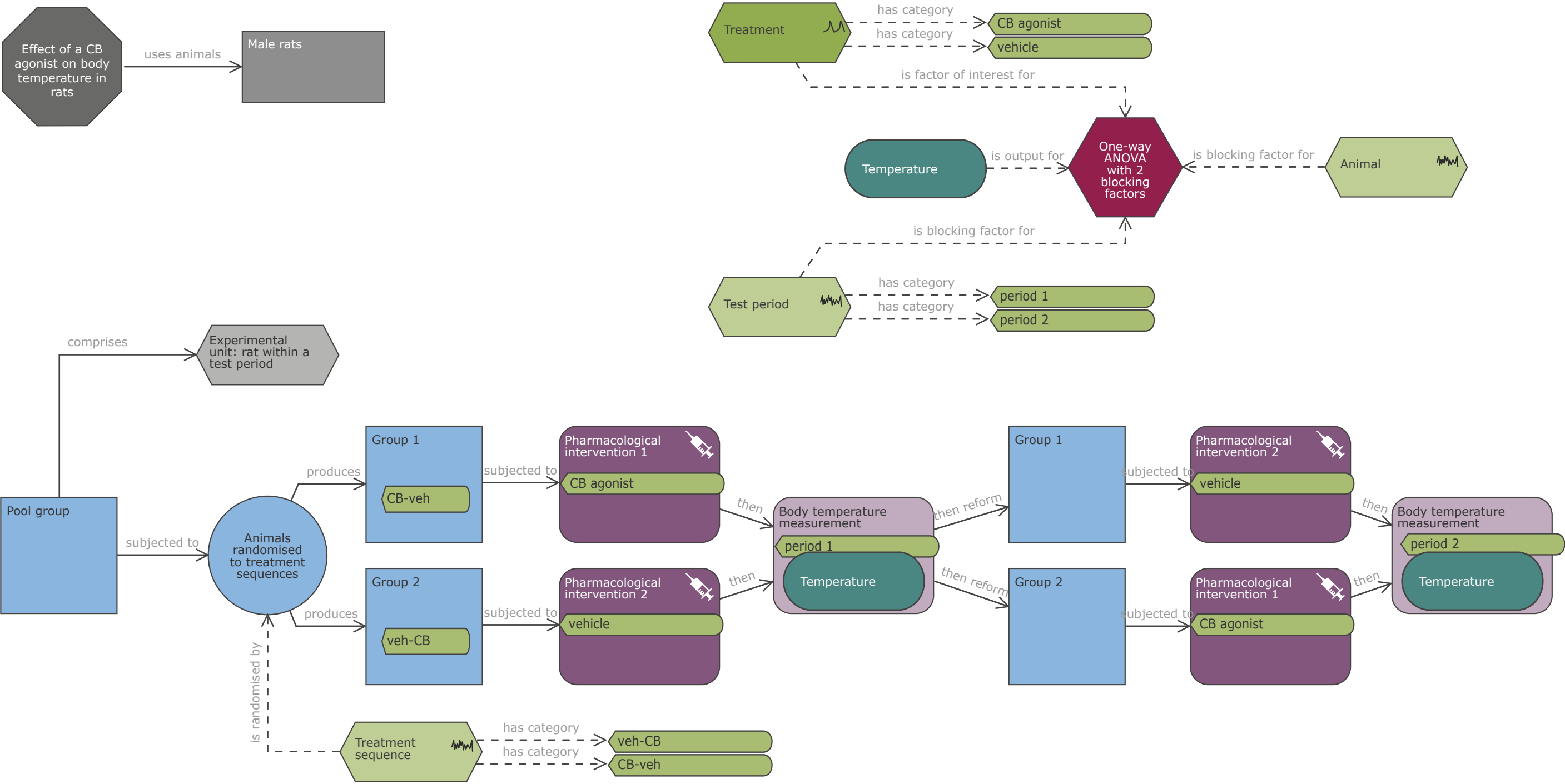

Diagram 2

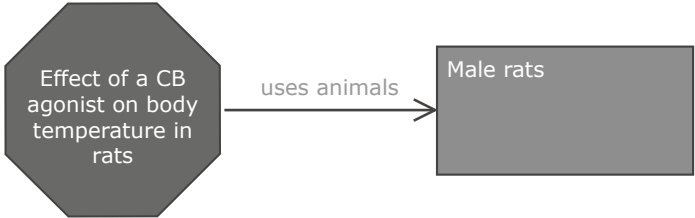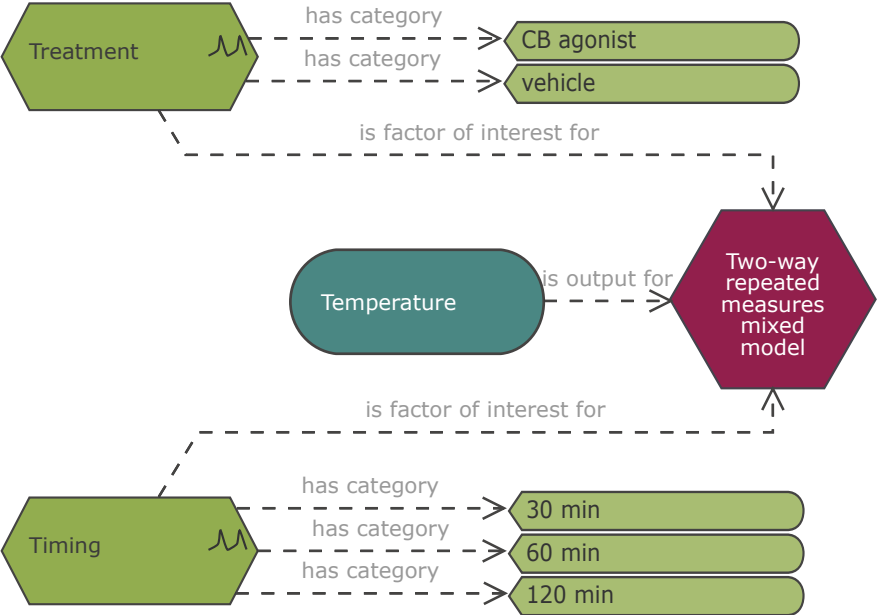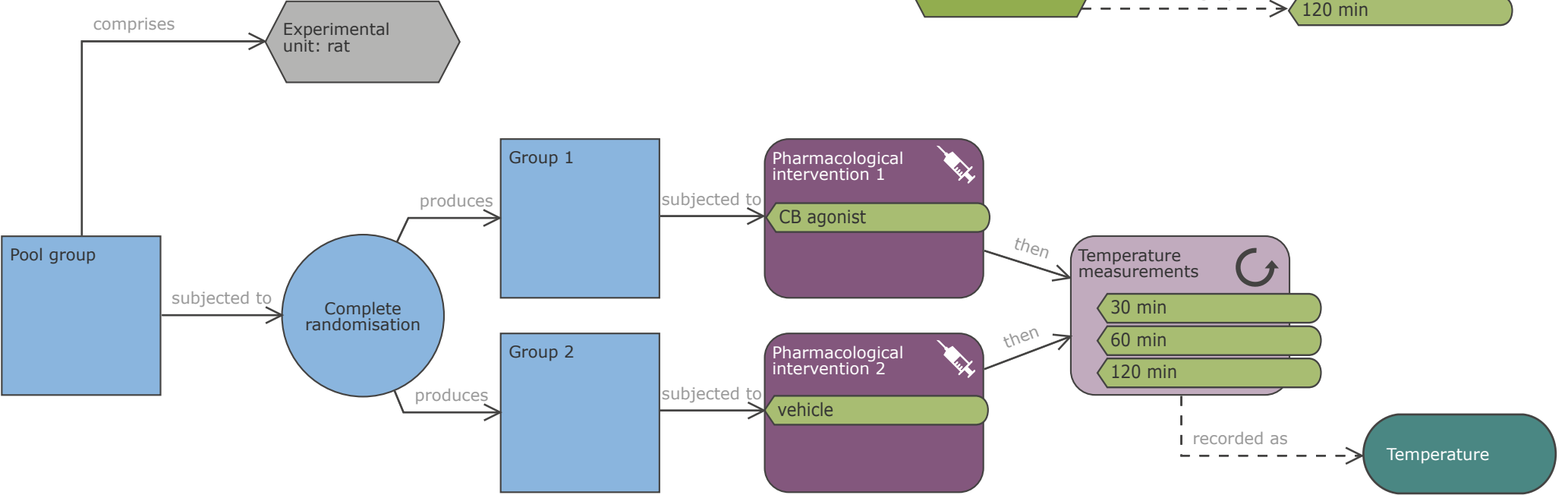

Diagram 3

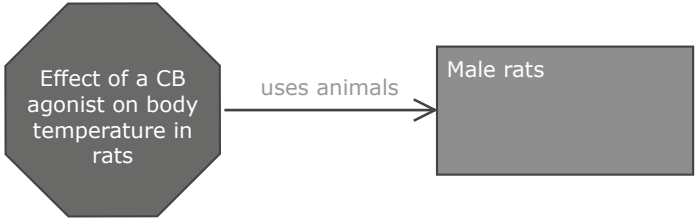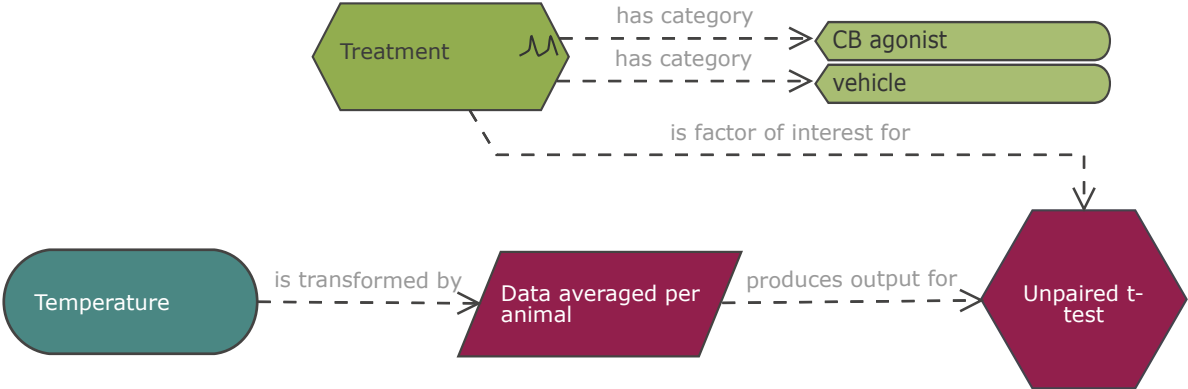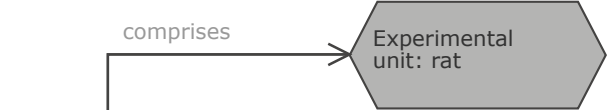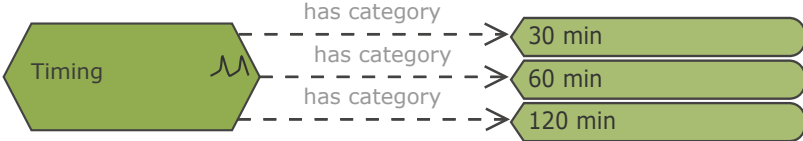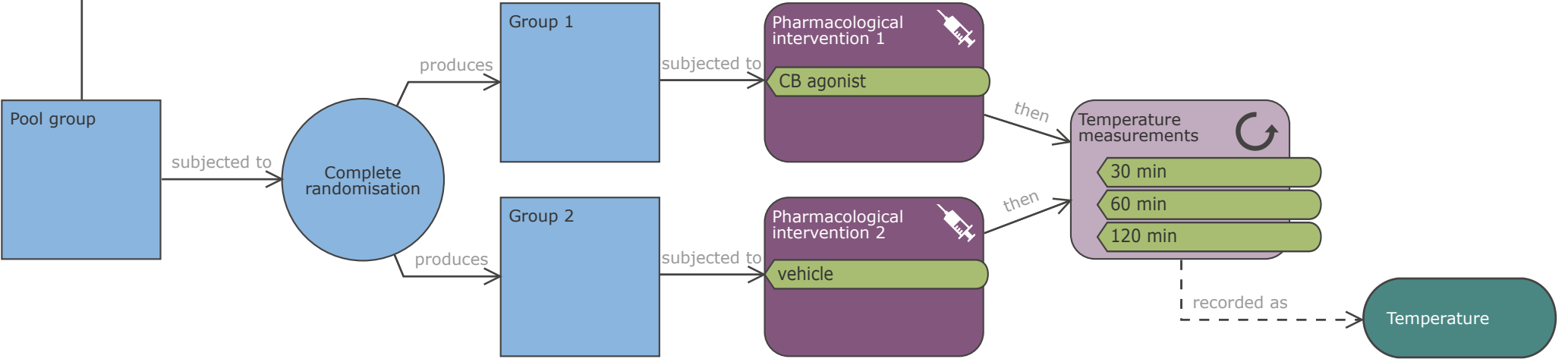

Diagram 4

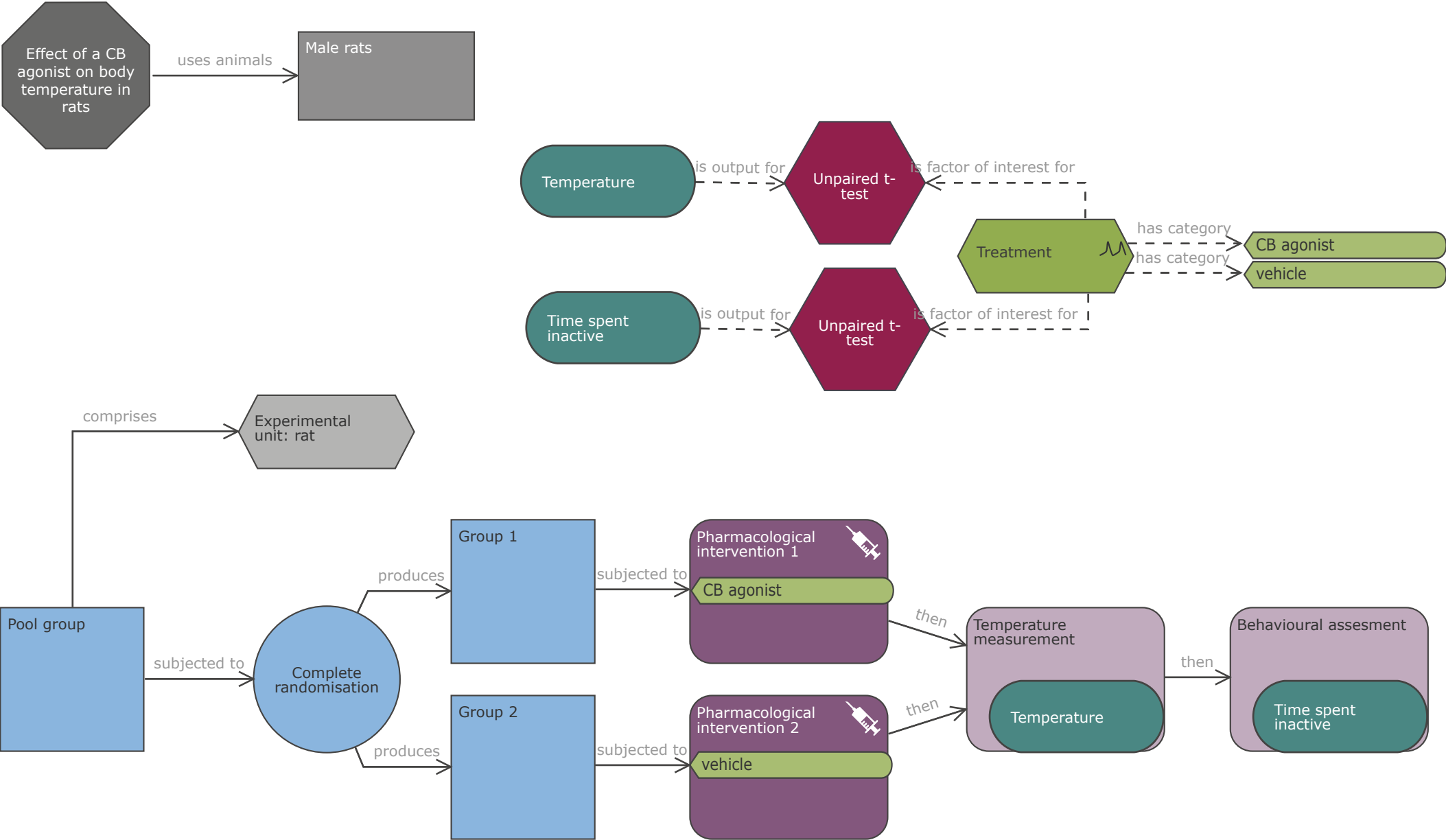

Supplement: S1 Fig — Diagram 1 shows an experiment in which animals receive multiple treatments in a different order for each animal, and each animal is used as its own control. Diagram 2 shows an experiment in which different groups of animals receive different treatments, 1 treatment per group, and the response to these treatments is measured over time, with each time point included in the analysis. Diagram 3 shows an experiment in which different groups of animals receive different treatments, 1 treatment per group, and the response to these treatments is measured over time, but a summary measure is taken for each animal. Diagram 4 shows an experiment in which multiple responses are measured for each animal. (PDF) [file pbio.2003779.s001.pdf]
